# Clinical and molecular spectrum of mucopolysaccharidosis IVA in Iraqi children: Allele-specific genotype–phenotype trends and novel GALNS variants

**DOI:** 10.1016/j.ymgmr.2025.101281

**Published:** 2025-11-27

**Authors:** Saja Baheer Abdul Wahhab, Rabab Farhan Thejeal

**Affiliations:** a Metabolic Clinic, Children Welfare Teaching Hospital, Medical City Complex, Baghdad, Iraq; b Department of Genetics, Sidra Medicine, Doha, Qatar; c College of Medicine, University of Baghdad – Pediatric Department, Pediatric Gastroenterology, Children Welfare Teaching Hospital, Medical City Complex, Baghdad, Iraq

**Keywords:** Mucopolysaccharidosis IVA, Morquio A syndrome, *GALNS* gene, Lysosomal storage disorders, Allele-specific genotype–phenotype trends, Next-generation sequencing, Skeletal dysplasia, Echocardiography, Glycosaminoglycans, Iraq

## Abstract

**Background:**

Mucopolysaccharidosis type IVA (MPS IVA; Morquio A syndrome) is a rare lysosomal storage disorder that causes early-onset skeletal dysplasia and progressive multisystem involvement. Although international cohorts have been described, population-specific data from the Middle East remain limited.

**Objective:**

This study aimed to characterize the clinical spectrum, functional status, and *GALNS* variant profile in Iraqi children with MPS IVA, and to assess diagnostic timing in comparison with international experience.

**Methods:**

We retrospectively analyzed 33 children from 26 unrelated families with confirmed MPS IVA, recruited at the Children's Welfare Teaching Hospital in Baghdad between 2016 and 2022. Enzyme activity was measured on dried blood spots by LC-MS/MS, and *GALNS* variants were identified using next-generation sequencing with Sanger confirmation. Clinical evaluation included anthropometry, echocardiography, ophthalmologic and audiologic assessments, skeletal surveys, and functional endurance testing (6-min walk test and 3-min stair-climb).

**Results:**

Fifteen distinct *GALNS* variants were identified, including five novel variants (three missense, two frameshift). The most common allele was c.949G > A (p.Gly317Arg), consistently associated with severe phenotypes and absent enzyme activity. Universal skeletal dysplasia was observed, with frequent corneal clouding, cardiac valve disease, and reduced functional endurance. Symptoms typically appeared in early childhood, but definitive diagnosis was often delayed until later childhood or adolescence. Allele-specific genotype–phenotype trends were observed, though no statistically significant correlations could be established due to limited cohort size.

**Conclusion:**

This first Iraqi cohort expands the *GALNS* variant spectrum and highlights persistent diagnostic delays despite early clinical manifestations. Early molecular confirmation, establishment of national registries, and multidisciplinary surveillance are essential to reduce long-term morbidity.

## 1 Introduction

Mucopolysaccharidosis type IVA (MPS IVA; Morquio A syndrome) is an autosomal recessive lysosomal storage disorder caused by deficiency of *N*-acetylgalactosamine-6-sulfatase (GALNS), an enzyme essential for the degradation of keratan sulfate and chondroitin-6-sulfate [1]. The enzymatic defect leads to progressive glycosaminoglycan (GAG) accumulation in multiple tissues, with skeletal dysplasia being the most prominent clinical feature [1,2]. Beyond the classical phenotype, increasing recognition of attenuated lysosomal storage disorders has broadened the clinical spectrum of MPS and related conditions; these milder forms often retain residual enzyme activity, present later, and manifest with organ-limited or nonspecific features that obscure early diagnosis [3].

The *GALNS* gene, located on chromosome 16, encodes a 522-amino-acid protein. To date, more than 350 unique variants have been reported worldwide, with missense variants accounting for the majority [4]. Zanetti et al. (2021) recently compiled data from 1190 patients, classifying 446 unique *GALNS* variants (68 novel); over 100 were strongly associated with severe phenotypes, while more than 30 correlated with attenuated disease [5], underscoring the condition's significant genetic heterogeneity.

MPS IVA demonstrates wide phenotypic variability, ranging from classic, rapidly progressive forms characterized by short stature, severe skeletal dysplasia, cervical instability, and early mortality, to attenuated forms with milder skeletal features and longer survival [[6], [7], [8]]. Key manifestations include growth impairment, genu valgum, kyphoscoliosis, pectus carinatum, joint laxity, corneal clouding, cardiac valve disease, respiratory compromise, and hearing loss, while cognitive function is typically preserved [1,6,9]. Recent studies have emphasized objective stratification of severity using height *Z*-scores, endurance tests such as the 6-min walk test, and radiographic scoring systems [7,8]. In attenuated presentations, isolated musculoskeletal, ophthalmic, cardiac, or airway findings may predominate, frequently leading to specialty-specific evaluations and a prolonged “diagnostic odyssey” [3,10].

Epidemiological data demonstrate significant geographic variation. The reported birth prevalence of MPS IVA ranges from approximately 1 in 76,000 in Northern Ireland to 1 in 640,000 in Western Australia [4]. A global update reviewing 33 countries confirmed these disparities and highlighted the influence of founder effects and consanguinity on regional frequencies [11]. Similarly, US registry-based data showed an incidence of ∼0.14 per 100,000 live births [12], considerably lower than Asian and Middle Eastern estimates, suggesting underdiagnosis in some populations. Borges et al. (2020) further suggested that the true prevalence may be higher than clinical reports indicate, based on population-scale genomic data [13].

Despite clinical recognition, diagnostic delays remain a global challenge. Infants are often asymptomatic at birth, but more than 70 % develop skeletal manifestations by early childhood [1,6]. International cohorts highlight years of delay between initial symptoms and definitive diagnosis; for example, the Malaysian national cohort reporting a median onset at 2.6 years and diagnosis at 6.9 years [14]. A Spanish adult cohort also revealed many patients were only diagnosed in adolescence or adulthood, despite early symptoms [8]. More recently, Yi et al. (2024) demonstrated that mildly affected patients may wait over five years from first symptoms to diagnosis, largely due to subtle orthopedic features [15]. Standard screening biomarkers (e.g., urinary GAGs) and even enzyme assays can be equivocal in attenuated disease, where borderline results and pseudodeficiency alleles may confound interpretation. This makes the early integration of next-generation sequencing a practical strategy to shorten time-to-diagnosis and enable cascade testing [3].

Management requires long-term multidisciplinary care, including orthopedic, cardiopulmonary, ophthalmologic, and audiologic surveillance [16]. Enzyme replacement therapy (ERT) with elosulfase alfa has demonstrated improvements in endurance, pulmonary function, and quality of life, though it remains limited in addressing skeletal pathology [[17], [18], [19]]. A 2022 regional consensus highlighted the need for standardized outcome measures and broader access to ERT across age groups [19]. Hematopoietic stem cell transplantation (HSCT) has also shown encouraging results, with recent pediatric data reporting normalized enzyme activity, improved growth, and functional gains [20]. Experimental gene therapy approaches have demonstrated correction of skeletal and systemic pathology in animal models, indicating promise for future translational therapies [21].

Taken together, the challenges of attenuated phenotypes, nonspecific presentations, and biomarker limitations contribute to persistent diagnostic delays internationally. In Iraq, where consanguinity is common and local data are lacking, the distribution of *GALNS* variants and the clinical spectrum remain undefined, limiting timely recognition and counseling [3]. Accordingly, this study was designed with three objectives: (i) to comprehensively characterize the clinical, radiographic, and functional features of a cohort of Iraqi children with MPS IVA, (ii) to define the underlying *GALNS* variant spectrum, including novel alleles, and (iii) to compare diagnostic timelines and phenotypic patterns with published international cohorts to assess implications for earlier detection and optimized multidisciplinary management.

## 2 Methods

### 2.1 Study design and participants

We conducted a retrospective, descriptive study of Iraqi children diagnosed with MPS IVA at the rare disease clinic of the Children's Welfare Teaching Hospital in Baghdad between 2016 and 2022. We included all patients with a confirmed molecular diagnosis of MPS IVA registered at the clinic during this period. A single investigator collected data from patient medical records using a standardized form to ensure consistency.

A total of 150 patients with various types of Mucopolysaccharidoses (MPS) were registered at our center during the study period. From his cohort, we focused on the 33 patients with genetically confirmed MPS IVA, representing 26 different families. No patients with only clinical suspicion (without genetic confirmation) were included.

### 2.2 Clinical and functional assessments

Data collected included demographic details, anthropometric measurements and results from ophthalmologic, audiologic, cardiac (echocardiography) and abdominal ultrasound evaluations. Clinical manifestations were identified and recorded based on definitions from recent literature [2,4,6]. Pedigrees were charted to assess family history and consanguinity rates, which were confirmed through interviews and considered positive if parents were first cousins or closer. Enzyme activity was measured using liquid chromatography– tandem mass spectrometry (LC-MS/MS) on dried blood spots analyzed at accredited international reference laboratories (Archimed Life Science, Vienna, Austria and Centogene AG, Rostock, Germany) [22]. Growth was assessed using MPS IVA-specific growth charts [6,9]; height and weight measurements were converted into *Z*-scores based on the Morquio A reference data. Ambulatory capacity was evaluated using the 6-min walk test (6-MWT) and 3-min stair-climb test per American Thoracic Society guidelines [23]. These functional Tests were performed only in ambulatory patients aged ≥5 years; results were stratified by age group (5–10, 11–16 years) and interpreted against normative data from MPS IVA natural-history studies [16,17].

### 2.3 *GALNS* variant analysis

Molecular genetic analysis was performed on all patients. Genomic DNA was extracted from dried blood spot. Targeted Next Generation Sequencing (NGS) was employed to analyze all coding exons and the flanking intronic (splice junction) regions of the *GALNS* gene at two accredited laboratories (Archimed, Vienna and Centogene, Rostock). Any regions with insufficient covering were re-analyzed by Sanger sequencing to ensure 100 % coverage. Variant nomenclature used the reference sequences NM_001323544.1 and NM_000512.4. Identified variants were classified according to ACMG/AMP guidelines [24].

### 2.4 Statistical analysis

Given the descriptive and retrospective design of this study, data were analyzed using descriptive statistical methods. Continuous variables (e.g., age at study, age at onset, height and weight *Z*-scores, enzyme activity, and 6-min walk distance) were summarized as mean ± standard deviation (SD) and range. Categorical variables (e.g., phenotype severity, presence of corneal clouding, cardiac valve disease) were presented as counts and percentages. For exploratory purposes, patients were grouped according to predicted molecular consequence: Group 1 (null/null genotypes, including frameshift or canonical splice-site variants) and Group 2 (missense or compound heterozygous genotypes). Mean height *Z*-scores and enzyme activity trends between these groups were compared descriptively to identify allele-specific genotype–phenotype patterns. Because of the limited sample size, no formal statistical testing software or *p*-value calculations were applied, and results are presented as descriptive trends only.

### 2.5 Ethics

The study was approved by the Ethics Committee of the Children Welfare Teaching Hospital (approval identifier: IRB SEP/2022/1) and complied with the Declaration of Helsinki [25]. Given the retrospective nature, patient data were de-identified. Informed consent was obtained from the parents or legal guardians for the use of medical data and publication of findings.

## 3 Results

Our study involved 33 patients from 26 distinct families. These patients were referred to our center from various provinces in Iraq:

• North: 10 families (15 patients)
• East: 3 families (3 patients)
• Middle: 11 families (13 patients)
• West: 1 family (1 patient)
• South: 1 family (1 patient)

A significant 91 % of the patients reported parental consanguinity. During the study, 12 out of the 33 patients began weekly enzyme replacement therapy.

Table 1 illustrates the patient demographics. The average age at enrollment was 8 years, with ages spanning from 1.9 to 16 years. Of the total, 11 were boys, accounting for 33.3 %. Symptoms typically manifested around 1.5 years of age, with approximately 60.6 % being diagnosed between their 1st and 3rd year. Diagnoses were made as early as 1 year and as late as 16 years, with a significant portion (50 %) being diagnosed between the ages of 5 to 12. Analysis of height *Z*-scores demonstrated significant growth impairment, with 93.9 % of individuals falling below the median (Z < 0). Notably, 72.8 % exhibited moderate to severe stunting (Z < −3), and 27.3 % were classified as severely stunted (Z < −5), reflecting substantial chronic malnutrition and halted linear growth. (Table 1).

**Table 1:** Patient demographics and diagnosis timeline.

Number included	*N* (%) 33 (100)	Boys 11 (33.3 %, girls 22 (66.6 %)
Age (years)	Mean ± standard deviation	8 ± 3.6
	Range	1.9–16
Age at manifestation (years)	Mean ± standard deviation	1.5 ± 0.9
	Birth-1st	8 (24.2 %)
	1st − 3rd	20 (60.6 %)
	>3	5 (15.2 %)
Age at diagnosis (years)	Range	1–16
	<3	4 (12.1 %)
	3–5	7 (21.2 %)
	5–12	17 (51.5 %)
	>12	5 (15.2 %)
Height Z score	Positive score	2 (6.1 %)
	0 to −3	7 (21.2 %)
	−3 to −5	15 (45.5 %)
	−5 and more	9 (27.3 %)
Weight Z score	Positive score	1 (3 %)
	0 to −3	6 (18.2 %)
	−3 to −5	13 (39.4 %)
	−5 and more	13 (39.4 %)

**Fig. 1:**
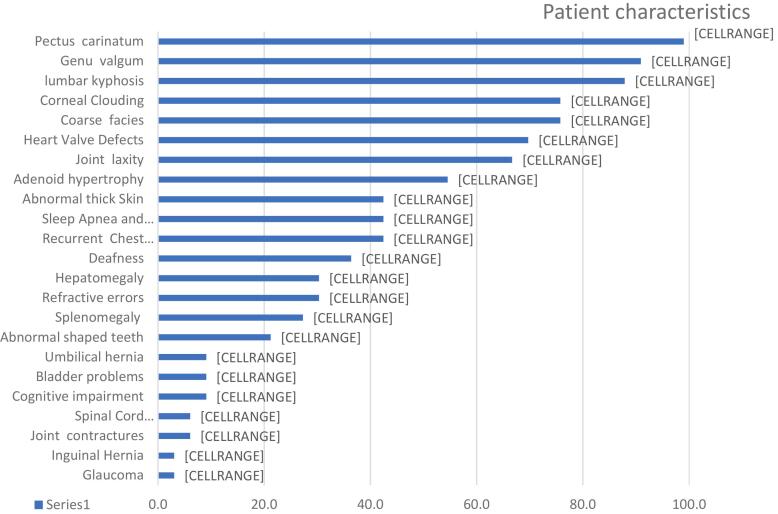
Overview of clinical characteristics and prevalence in patients with mucopolysaccharidosis IVA.

The clinical characteristics of the patients are summarized in Fig. 1. Mild facial coarseness was observed in 75 % of the patients, while 25 % exhibited no signs of facial coarseness. Joint laxity and heart valve defects were present in approximately two-thirds of the patients, with prevalence rates of 66.7 % and 69.7 %, respectively. The predominant valvular abnormalities included mitral valve prolapse, thickening, and mitral regurgitation. At the time of examination, 75.8 % of the patients had corneal clouding, and deafness was reported in 36.4 % of cases. Pectus carinatum was seen in all patients, followed by genu valgum in 90.9 % and kyphosis in 87.9 %.

**Fig. 2:**
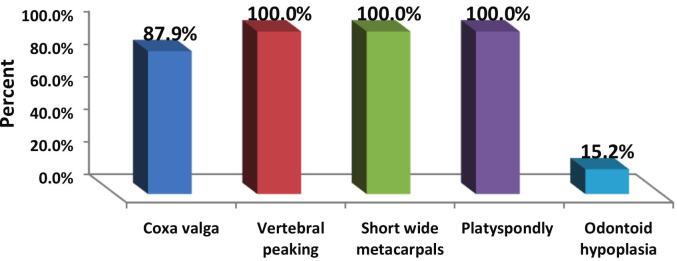
Prevalence of major skeletal features in patients with MPS IVA, including coxa valga, vertebral beaking, short metacarpals, platyspondyly, and odontoid hypoplasia.

Regarding skeletal changes on radiography, Fig. 2 displayed that all the patients had platyspondyly, vertebral peaking and short wide metacarpals, while 87 % had coxa valga. Odontoid hypoplasia was identified in 15 % only.

**Fig. 3:**
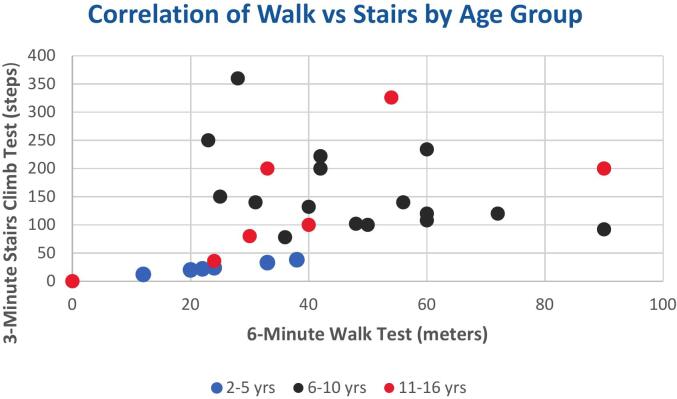
Scatterplot illustrating the correlation between the 6-min walk test (6-MWT, meters) and the 3-min stair climb test (steps), stratified by age group. Blue = 2–5 years, black = 6–10 years, and red = 11–16 years. The plot demonstrates the relationship between functional endurance and lower-limb strength across pediatric age groups.

To evaluate physical endurance as in Fig. 3, participants underwent the 6-min walk test (6-MWT, meters) and the 3-min stair-climb test (steps) following American Thoracic Society guidelines [23]. Ambulatory patients across all age groups were included, provided they could complete the tests safely; non-ambulatory individuals were excluded from analysis. In total, 31 valid data points were analyzed. Results were stratified into three age groups (2–5, 6–10, and 11–16 years) to account for developmental differences in endurance and muscle strength. The mean performance was 43.6 steps (SD ± 20.8) for the stair-climb test and 157.2 m (SD ± 74.2) for the 6-MWT. This stratified approach allowed appropriate comparison of functional capacity across pediatric age groups. When stratified by age, mean 6-min walk test (6-MWT) distances were 118 ± 42 m for children aged 2–5 years, 162 ± 63 m for 6–10 years, and 189 ± 68 m for 11–16 years.

Corresponding mean 3-min stair-climb counts were 29 ± 9, 44 ± 14, and 53 ± 17 steps, respectively. Despite this age-related improvement, performance in all groups remained substantially below normative values reported in natural-history studies [16,17].

As shown in Fig. 3, there was a positive correlation between the 6-min walk test (6-MWT) distance and the 3-min stair climb test (steps) across all age groups. Children in the 2–5 years group clustered at lower walk distances and stair counts, while those in the 6–10 years group showed intermediate performance. Participants aged 11–16 years generally demonstrated higher stair counts despite some variability in walk test performance. This distribution highlights age-related increases in functional endurance and muscle strength. It is important to interpret 6-MWT results in the context of age and ambulation status. Even when restricted to older, ambulatory patients, distances remained far below normative values, consistent with previous natural-history studies demonstrating severe endurance limitations in MPS IVA [16,17].

### 3.1 *GALNS* gene molecular analysis

Next-generation sequencing of the *GALNS* gene identified fifteen distinct variants, including five novel variants (three missense and two frameshift). Most variants were missense (11/15), followed by two frameshift and two splice-site variants. Two patients carried compound heterozygous genotypes.

Patient #3 had *c.188C* *>* *G (p.Ala63Gly)* and *c.1265 A >* *G (p.Gln422Arg)*, both classified as likely pathogenic and associated with an attenuated phenotype. Patient #22 carried *c.230C* *>* *G (p.Pro77Arg)* and *c.433C* *>* *T (p.His145Tyr)*, both previously reported pathogenic variants. The remaining 31 patients were homozygous for their respective variants (Table 2).

**Table 2:** Clinical, biochemical, and genetic features of 33 *Mucopolysaccharidosis IVA* patients (*n* = 33).

Patient ID	Family ID	Sex	AS	AO	AD	Height	Phenotype	EA	Nucleotide change	Protein change
1	1	Female	3	0.3	1	83	Severe^a^	0.8	c.433C > T	p.His145Tyr
2	2	Female	15.2	2	13	88	Severe	1.2	c.145-146del	p.Tyr49Trpfs*108
3	3	Female	5.3	3	4	93	Severe	1.4	c.188C > G/c.1265 A > G^¥^	p.Ala63Gly/p.Gln422Arg
4	4	Female	8.2	2	8	85.5	Severe	0.00	c.949G > A	p.Gly317Arg
5	4	Female	9.4	1	9	95	Severe	0.00	c.949G > A	p.Gly317Arg
6	5	Male	16	1.5	16	89	Severe	0.00	c.949G > A	p.Gly317Arg
7	6	Female	8.4	1.5	8	93	Severe	0.00	c.949G > A	p.Gly317Arg
8	7	Male	6.6	1	7	85	Severe	0.00	c.949G > A	p.Gly317Arg
9	7	Male	6.6	1	7	82	Severe	0.00	c.949G > A	p.Gly317Arg
10	8	Female	10.2	2	10	89	Severe	0.00	c.949G > A	p.Gly317Arg
11	8	Female	7.2	2	6	83	Severe	0.00	c.949G > A	p.Gly317Arg
12	9	Female	14.2	1	12	93	Severe	0.00	c.433C > T	p.His145Tyr
13	9	Female	12	3	10	91	Severe	0.00	c.433C > T	p.His145Tyr
14	10	Female	5.6	1	4	86.5	Severe	0.8	c.1199G > C	p.Ala400Pro
15	11	Male	9.6	1.5	7	95	Severe	1.2	c.949G > A	p.Gly317Arg
16	12	Female	3.9	0.4	3	85	Severe	0.9	c.899-1G > C	p.?
17	12	Female	5	0.5	5	90	Severe	1.4	c.899-1G > C	p.?
18	12	Male	7.9	0.6	8	86	Severe	0.8	c.899-1G > C	p.?
19	13	Female	7.9	1	6	94	Severe	1.5	c.949G > A	p.Gly317Arg
20	14	Male	10.5	2	10	87	Severe	0.00	c.415G > A	p.Gly139Ser
21	15	Female	3.2	1	3	87	Severe	0.00	c.196G > C	p.Gly66Arg
22	16	Male	8.8	1	9	96	Severe	0.00	c.230C > G/c.433C > T^¥^	p.Pro77Arg/p.His145Tyr
23	17	Male	13.5	0.6	12	92	Severe	0.00	c.230C > G	p.Pro77Arg
24	18	Female	9	1.6	8	93	Severe	1.2	c.949G > A	p.Gly317Arg
25	19	Male	9	3	4	98	Severe	0.00	c.1218_1221dupCAAC	p.Ser408GlnfsTer11
26	19	Female	4	3	4	87	Severe	0.00	c.1218_1221dupCAAC	p.Ser408GlnfsTer11
27	20	Male	4.2	0.8	1.1	87	Severe	0.1	c.775C > T	p.Arg259Trp
28	21	Female	6.5	3	4	93	Severe	0.00	c.120 + 1G > C	p.?
29	22	Male	6.8	2.6	5.6	106.5	Attenuated^€^	0.00	c.244 T > C	p.Ser82Pro
30	23	Female	1.9	0.8	1.8	75	Severe	0.00	c.374C > T	p.Pro125Leu
31	24	Female	6.3	1	1.5	89	Severe	0.00	c.1218_1221dupCAAC	p.Ser408GlnfsTer11
32	25	Female	6	2	5	86	Severe	0.2	c.433C > T	p.His145Tyr
33	26	Female	13	0.5	13	110	Severe	0.00	c.1265A > G	p.Gln422Arg

Group 1 (null/null) = frameshift or canonical splice-site variants (patients #2, #16–18, #25–26, #28, #31). Group 2 (missense ± compound het) = remaining 25 patients. Overall height *Z*-score distribution for the cohort is summarized in Table 1.

a Classification based on Morqio growth chart. ^¥^ compound heterozygous ^€^ Attenuated as in less severe phenotype of MPS IVA AD: Age at diagnosis (year). Ala: Alanine. AO: Age of onset (year). Arg: Arginine. AS: Age at study (year). EA: Enzyme activity (umol/l/h). Gln: Glutamine. Gly: Glycine. His: Histidine. Pro: Proline. Ser: Serine. Tyr: Tyrosine.

The most frequent allele was *c.949G* *>* *A (p.Gly317Arg)*, detected in eleven patients. One missense variant, *c.244* *T* *>* *C (p.Ser82Pro)* in exon 2, was classified as a variant of uncertain significance (VUS) and was associated with a milder (attenuated) phenotype. This patient exhibited mild skeletal features, no hearing loss or corneal clouding, normal echocardiographic findings, and no organomegaly, despite undetectable enzyme activity. Urinary glycosaminoglycan (GAG) quantification was unavailable at the time of testing.

The amino-acid substitutions in the compound heterozygous patient (Ala→Gly at position 63 and Gln → Arg at position 422) are located at conserved residues and were predicted deleterious by several *in-silico* algorithms (PolyPhen-2, SIFT, MutationTaster) (Table 3).

**Table 3:** MPS IVA patients with known and novel *GALNS* variants identified in this study.

Chromosome: position	Nucleotide change; protein change	Effect	Zygosity	Number of patients	ClinVar ID	ClinVar classification	References
16: 88904163	c.433C > T; His145Tyr	Missense	Homozygous	4	VCV001048457.3	Likely pathogenic	Morrone et al. 2014 https://doi.org/10.1002/humu.22635
16: 88898459	c.949G > A; Gly317Arg	Missense	Homozygous	11	VCV001048309.9	Conflicting interpretations of pathogenicity	Pollard et 2013 https://doi.org/10.1007/s10545012-9533-7
16: 88909162	c.196G > C; Gly66Arg	Missense	Homozygous	1	VCV001048268.3	Uncertain significance	Laradi et al. 2006 https://doi.org/10.1016/j.ymgme. 2005.11.001
16: 88907407	c.415G > A; Gly139Ser	Missense	Homozygous	1	VCV000265167.33	Pathogenic	Tomatsu et al. 1997. http://doi.org/10.1002/(SICI)1098–1004(1997)10:5≤368::AIDHUMU6≥3.0.CO;2-B
16: 88909128	c.230C > G; Pro77Arg	Missense	Homozygous	2	VCV000856567.14	Pathogenic/Likely pathogenic	Tomatsu et al. 1995 https://doi.org/10.1093/hmg/4.4.741
16: 88909114	c.244 T > C; Ser82Pro	Missense	Homozygous	1	VCV001048391.3	Uncertain significance	Dung et al. 2013 https://doi.org/10.1016/j.ymgme.2013.06.008
16: 88907448	c.374C > T; Pro125Leu	Missense	Homozygous	1	VCV000873151.12	Pathogenic/Likely pathogenic	Tomatsu et al. 1997. http://doi.org/10.1002/(SICI)1098-1004(1997)10:5≤368::AIDHUMU6≥3.0.CO;2-B
16: 88898510	c.899-1G > C	Splice acceptor	Homozygous	3	VCV001048482.4	Pathogenic	Carraresi et al. 2008 https://doi.org/10.1016/j.cca.2008.07.021 Xiong et al. 2015 https://doi.org/10.1126/science.1254806
16: 88923165	c.120 + 1G > C	Splice donor	Homozygous	1	VCV000847816.12	Pathogenic	Bidchol et al. 2014 https://doi.org/10.1002/ajmg.a.36735
16: 88901744	c.775C ≥ T; p. Arg259Trp	Missense	Homozygous	1	VCV001675645.8	Likely pathogenic	

*MPS IVA patient with novel GALNS variants*					
NM_000512.5	c.1218_1221dupCAAC; Ser408GlnfsTer11	Frameshift	Homozygous	3	It was classified as pathogenic (Class 1) by Archimed Life Science GmbH, Vienna, according to ACMG/AMP criteria (PVS1 + PM2). This homozygous frameshift creates a premature stop codon predicted to cause loss of function via nonsense-mediated decay, consistent with the established pathogenic mechanism of *GALNS* deficiency.
NM_000512.5	c.1265A > G; Gln422Arg	Missense	Homozygous	1	It was reported as pathogenic (Class 1) by Archimed Life Science GmbH, based on absence from population databases and consistent pathogenic in-silico predictions (PM2 + PP3 + PP4). The homozygous state supports its diagnostic relevance for MPS IVA.
NM_000512.5	c.145-146del; Tyr49Trpfs*108	Frameshift	Homozygous	1	The *GALNS* frameshift variant c.145_146del (p.Tyr49Trpfs*108) (equivalent to c.164_165del based on NM_001323544.1) was identified in a homozygous state. The deletion introduces a premature stop codon 108 residues downstream, predicted to cause nonsense-mediated decay. This variant was classified as pathogenic (Class 1) by Centogene AG based on ACMG/AMP criteria (PVS1 + PM2).
NM_000512.5	c.188C > G; Ala63Gly	Missense	Heterozygous	1	This missense substitution affects a highly conserved alanine residue and was predicted to be deleterious by multiple in silico tools (PolyPhen-2: probably damaging; SIFT: deleterious; MutationTaster: disease causing). Centogene AG classified this variant as likely pathogenic (Class 2) based on ACMG/AMP criteria PM2 + PP3 + PP4, considering its absence from population databases and consistency with the patient's biochemical and clinical phenotype of MPS IVA.
NM_000512.5	c.1199G > C; Ala400Pro	Missense	Homozygous	1	This missense change substitutes a highly conserved alanine residue with proline within the catalytic domain of the enzyme. In-silico prediction tools supported a deleterious effect (PolyPhen-2: probably damaging; SIFT: deleterious; MutationTaster: disease causing), and the residue was highly conserved across species. Centogene AG classified this variant as likely pathogenic (Class 2) according to ACMG/AMP guidelines (PM2 + PP3 + PP4)

To explore possible genotype–phenotype trends, patients were descriptively grouped according to variant type and predicted molecular consequence. Group 1 (*n* = 8) carried *null* alleles (frameshift or canonical splice-site variants, predicted loss-of-function), while Group 2 (*n* = 25) carried *missense* variants (residual enzyme activity predicted).

Patients with *null/null* genotypes exhibited the lowest height *Z*-scores (mean − 5.4 ± 0.9) and were all classified as severe, whereas those carrying *missense/missense* or *compound heterozygous* genotypes showed milder skeletal restriction (mean height Z-score − 3.2 ± 0.8) and greater residual enzyme activity.

The most frequent allele, *c.949G* *>* *A (p.Gly317Arg)*, was consistently associated with severe short stature and absent enzyme activity, supporting prior reports of its strong correlation with classical Morquio A phenotype (Pollard et al., 2013). In contrast, the variant *c.244* *T* *>* *C (p.Ser82Pro)* was found in a patient with a milder (attenuated) phenotype, consistent with residual functional enzyme or partial activity.

Although no statistically significant associations could be demonstrated across the entire cohort due to small group sizes, allele-specific trends were evident: frameshift and splice-site variants consistently produced severe disease, while some *missense* alleles were associated with intermediate or attenuated forms. These descriptive findings reflect allele-specific genotype–phenotype trends rather than statistically proven correlations, consistent with the limited sample size. (Table 3, Fig. 3).

## 4 Discussion

Our study of 33 Iraqi patients with MPS IVA revealed high rates of consanguinity (91 %) and a predominance of severe phenotypes, consistent with the autosomal recessive inheritance and known impact of founder effects in Middle Eastern populations [13]. The universal presence of skeletal dysplasia and frequent multisystem involvement mirror the classical clinical spectrum of Morquio A described in international literature [2,6].

### 4.1 Manifestation age of MPS IVA

The mean age of symptom onset in our cohort was 1.5 years, aligning with GeneReviews and registry data describing onset in early childhood for severe phenotypes [2,4]. However, half of our patients were not diagnosed until after age 5, reflecting substantial diagnostic delay. Similar delays have been reported in the Malaysian cohort, where median diagnosis occurred 4 years after symptom onset [14], and in Yi et al. (2024), who found that mildly affected patients often faced >5 years of delay [15]. These findings highlight the global challenge of timely recognition, particularly for attenuated phenotypes with subtle presentations.

### 4.2 Clinical manifestation and phenotypes of MPS IVA

The pattern of clinical manifestations in our cohort—prominent skeletal deformities, corneal clouding, and cardiac valve disease—parallels those reported in international series. The Spanish experience emphasized bone dysplasia and intermediate to severe skeletal features in most patients [8]. Similarly, natural history studies [6,9,16] confirm that orthopedic and skeletal complications dominate disease burden, while extra-skeletal features such as corneal opacity and valvular disease further compromise quality of life. While attenuated forms have been described, most of our patients clustered toward the severe phenotype, which is in keeping with the high frequency of homozygous deleterious variants observed in populations with high consanguinity.

### 4.3 GALNS gene

Molecular analysis revealed 15 variants in *GALNS*, including five novel alleles, most of which were missense. The recurrent c.949G > A (p.Gly317Arg) allele was especially frequent and consistently associated with severe disease and absent enzyme activity in our cohort. Although ClinVar lists this allele as of “uncertain significance” [23], its recurrence and clear phenotype correlation in our study support pathogenicity, echoing previous registry-based analyses [5]. Frameshift alleles such as c.1218_1221dupCAAC further expand the mutational spectrum. The predominance of homozygous variants reflects the high consanguinity rate in Iraq, in line with genetic epidemiology of autosomal recessive diseases in the region. Our findings also mirror global observations of founder effects shaping the genetic landscape of MPS IVA. While formal statistical correlations could not be established due to cohort size limitations, descriptive grouping by variant type (null vs missense) revealed allele-specific trends consistent with disease severity.

For example, Indian cohort identified a recurrent *GALNS* variant (c.1218_1221dupCAAC) in multiple unrelated Indian families, strongly associated with a severe phenotype and absent enzyme activity [26]. We detected the same duplication variant in two unrelated Iraqi families, suggesting a possible founder effect or shared ancestral haplotype across regional populations. These parallels highlight the importance of population-specific variant databases and targeted molecular screening strategies, particularly in high-consanguinity settings, to improve diagnostic efficiency and enable cascade testing.

### 4.4 Growth and functional impairment

Growth assessment using MPS IVA–specific charts demonstrated profound impairment, with most patients far below age-matched norms. This observation is consistent with international natural history studies [6,16], which identify short-trunk dwarfism as a hallmark of Morquio A. Functional endurance, assessed through 6-MWT and 3-min stair climb, was also markedly reduced, mirroring findings from multinational registries [9]. Our observations regarding the early onset of clinical manifestations and pronounced growth restriction are in strong agreement with findings from East Asian studies. Chuang et al. [27] demonstrated that the majority of individuals with classical MPS IVA present within the first two years of life and show marked impairment in growth trajectory, often dropping below −3 SD during early childhood — trends that closely parallel those seen in our cohort. Moreover, their longitudinal analysis indicated that diagnostic delays remain frequent, with many patients going undiagnosed for several years despite the presence of early skeletal abnormalities, reflecting the diagnostic latency identified in our study. These parallels highlight the persistent need for increased clinical vigilance and earlier diagnostic evaluation in regions with high disease burden and elevated consanguinity rates.

### 4.5 Implications for diagnosis and therapy

Although our study did not evaluate treatment outcomes, the young age at symptom onset and severity of growth impairment underscores the importance of timely diagnosis and intervention. Lin et al. [28] highlighted that coordinated care — including early orthopedic intervention, cardiopulmonary surveillance, and enzyme replacement therapy — significantly improves functional outcomes and quality of life, even in patients with advanced disease. Incorporating such structured management strategies into clinical practice in Iraq could mitigate disease burden and enhance long-term outcomes for affected individuals. Evidence from clinical trials demonstrates that early initiation of enzyme replacement therapy (ERT) with elosulfase alfa improves endurance and pulmonary function [7,17,18], while regional consensus statements emphasize standardized monitoring and wider access [19]. Hematopoietic stem cell transplantation (HSCT) has also shown promising results, with recent pediatric outcomes indicating normalization of enzyme activity and improved growth [20].

These findings, together with international reports, strengthen the rationale for newborn screening (NBS). Pilot programs using tandem mass spectrometry have successfully included MPS IVA in expanded LSD screening panels [22]. In high-consanguinity populations such as Iraq, NBS could enable presymptomatic diagnosis, timely initiation of ERT or HSCT, and improved long-term outcomes.

Our findings are consistent with and further support recent regional recommendations. The 2024 Saudi Arabian consensus on MPS IVA [29] underscores the importance of early molecular diagnosis, structured multidisciplinary care, and timely initiation of disease-specific therapies — priorities that closely reflect the needs of our Iraqi cohort, characterized by high consanguinity and significant diagnostic delays. The consensus also calls for standardized follow-up protocols, growth and functional assessments, and the integration of newborn screening in high-risk populations. Implementing these strategies in Iraq could help close diagnostic gaps, improve access to enzyme replacement therapy, and guide decisions regarding hematopoietic stem cell transplantation. Moreover, incorporating MPS IVA into regional newborn screening programs could enable presymptomatic detection, allow earlier therapeutic intervention, and reduce long-term disease burden. Together, these recommendations highlight the need for coordinated national policies that embed genomic diagnostics, early treatment, and comprehensive care into rare disease management.

### 4.6 Limitations

This study has several limitations. Its retrospective design and reliance on hospital records limited some clinical and biochemical assessments. The absence of urinary GAG analysis reflects resource constraints. The sample size, though substantial for a single center in Iraq, remains modest compared to international registries. Finally, treatment outcomes were not evaluated, and prospective follow-up will be essential to assess the impact of ERT and other interventions.

## 5 Conclusion

We identified five novel *GALNS* variants and confirmed the predominance of severe phenotypes in Iraqi patients with MPS IVA. High consanguinity and diagnostic delay remain major challenges. These findings expand the global variant spectrum and underscore the urgent need for newborn screening and earlier access to therapies in resource-limited, high-consanguinity settings.

## CRediT authorship contribution statement

**Saja Baheer Abdul Wahhab:** Writing – review & editing, Writing – original draft, Resources, Project administration, Methodology, Investigation, Funding acquisition, Formal analysis, Data curation, Conceptualization. **Rabab Farhan Thejeal:** Visualization, Validation, Supervision, Software, Project administration.

## Ethics approval and consent to participate

The Ethics Committee of the Children Welfare Teaching Hospital granted ethical approval for this study (IRB: SEP/2022/1). All procedures adhered to the Declaration of Helsinki guidelines. Parents or legal guardians of the patients provided written consent for publication and any associated images.

## Funding

This research received no specific grant from any funding agency in the public, commercial or not-for-profit sectors.

## Declaration of competing interest

The authors declare that the research was conducted in the absence of any commercial or financial relationships that could be construed as a potential conflict of interest.

## Data Availability

The datasets used and/or analyzed during the current study are available from the corresponding author on reasonable request.
